# Innate and adaptive abnormalities in youth with vertically acquired HIV through a multicentre cohort in Spain

**DOI:** 10.1002/jia2.25804

**Published:** 2021-10-20

**Authors:** Itzíar Carrasco, Laura Tarancon‐Diez, Elena Vázquez‐Alejo, Santiago Jiménez de Ory, Talía Sainz, Miren Apilanez, Cristina Epalza, Sara Guillén, José Tomás Ramos, Cristina Díez, Jose Ignacio Bernardino, José Antonio Iribarren, Angielys Zamora, María Ángeles Muñoz‐Fernández, María Luisa Navarro, the Pediatric National AIDS Research Network of Spain (CoRISpe) integrated in the Translational Research Network in Pediatric Infectious Diseases (RITIP)

**Affiliations:** ^1^ Infectious Diseases in Paediatric Population Gregorio Marañón Research Institute (IiSGM) and University Hospital Madrid Spain; ^2^ Universidad Complutense de Madrid Madrid Spain; ^3^ Immunology Section Inmuno‐Biology Molecular Laboratory, Gregorio Marañón University General Hospital (HGUGM), Gregorio Marañón Health Research Institute (IiSGM), Spanish HIV HGM BioBank Madrid Spain; ^4^ Department of Paediatrics La Paz Research Institute (IdiPAZ) and University Hospital Madrid Spain; ^5^ Department of Paediatrics Donostia University Hospital País Vasco Spain; ^6^ Department of Paediatrics 12 de Octubre University Hospital Madrid Spain; ^7^ Department of Paediatrics Getafe University Hospital Madrid Spain; ^8^ Department of Paediatrics Clínico San Carlos University Hospital Madrid Spain; ^9^ Department Infectious Diseases Gregorio Marañón Research Institute and University Hospital Madrid Spain; ^10^ Department of Infectious Diseases La Paz Research Institute (IdiPAZ) and University Hospital Madrid Spain; ^11^ Department of Infectious Diseases Donostia University Hospital País Vasco Spain; ^12^ Biochemistry Section Gregorio Marañón University Hospital Madrid Spain

**Keywords:** activation, antiretroviral treatment (ART), exhaustion, HIV, immunosenescence, vertical transmission

## Abstract

**Introduction:**

Immune abnormalities have been described among youth with vertically acquired HIV (YWVH) despite antiretroviral treatment (ART). The CD4/CD8 ratio could be a useful prognostic marker. We assess immune activation and senescence in a cohort of YWVH in comparison to youth without vertically acquired HIV.

**Methods:**

YWVH under suppressive ART were included and compared to a group of HIV‐negative donors (HD) matched by age and sex, from September 2019 to September 2020. Subset distribution and expression of activation, maturation, senescence and exhaustion markers on T and NK cells were studied on peripheral blood mononuclear cells by multiparametric flow cytometry.

**Results:**

Thirty‐two YWVH (median age: 24.4 years (interquartile range: 22.5 to 28.3 years)) were included. Among YWVH, CD4‐ and CD8‐T cells showed high levels of activation (HLA‐DR/CD38), IL‐7 receptor expression (CD127) and exhaustion (TIM‐3). Regarding NK cells, YWVH showed increased levels of activation and exhaustion markers compared to HD. Strong inverted correlations were observed between T‐cell activation (HLA‐DR/CD38), senescence (CD57) and exhaustion (TIGIT, PD‐1) levels with the CD4/CD8 ratio among YWVH. HLA‐DR, CD69, NKG2D and NKG2A expression levels on NK cells also correlated with the CD4/CD8 ratio. Age at ART initiation was directly associated with higher frequency of CD16^high^ NK‐cell subsets, exhaustion T‐cell levels (CD57, TIM3) and NK cells activation levels.

**Conclusions:**

Immunological changes associated with vertically acquired HIV, characterized by increased activation and exhaustion levels in innate and adaptive immune components, are only partially restored by ART. The CD4/CD8 ratio can be a useful marker of disease progression for routine clinical practice.

## 1 INTRODUCTION

Since the availability of effective antiretroviral treatment (ART), HIV infection has become a chronic disease [1]. Although a cure is yet unachievable, ART has increased the life expectancy, decreasing morbidity and mortality by inhibiting viral replication. Studies have shown how ART decreases systemic inflammation and immune activation in people living with HIV (PLWH), even if it does not achieve fully restoration viral suppression is nonetheless maintained over time [2, 3, 4]. This fact is worrisome especially among youth with vertically acquired HIV (YWVH) [5, 6], exposed to the virus and its treatment since birth. According to the literature, this chronic immune dysfunction together with a proinflammatory environment fuels the appearance of comorbidities in PLWH [5, 7, 8], including cancer, cardiovascular diseases, osteoporosis, metabolic syndrome and liver related problems; diseases usually associated with ageing [9, 10]. Somehow, the altered immunity and chronic inflammation observed in PLWH have been compared to changes associated with ageing among the elderly, which seem to appear in a premature way in YWVH including children [11, 12, 13, 14].

Vertically acquired HIV infection is characterized by a higher viral load and a faster disease progression, maybe due to an immature immune system at the moment of infection [15, 16]. Even an early onset of ART in children, which prevents disease progression, does not guarantee a full restoration of the immune system. Although young age could be considered as an advantage due to the large immune resources and lymphocyte regeneration capacity [17], children infected at birth or *in utero* are characterized by a deficient immune response after immunization [15, 18]. It is still unclear whether the effects of the infection on an immune system under development are reversible later in life, as the first group of children infected since birth are now reaching adulthood. Immunological data on this population remain scarce, but a premature immune ageing profile associated with HIV infection has been described [11, 19, 20]. Identification of patients at risk for non‐AIDS‐related issues is capital for this population group, and the CD4/CD8 ratio has been suggested as a valuable marker for routine clinical practice [13].

The objective of this study was to evaluate the impact of HIV infection on the innate and adaptive immune system of ART‐treated YWVH, and to address the relationship between clinical parameters and the CD4/CD8 ratio and markers of immune activation, maturation and exhaustion.

## 2 METHODS

### 2.1 Study participants

A retrospective study including YWVH participating in the Paediatric Spanish AIDS Research Network Cohort (CoRISpe) was performed [21]. All patients were on stable ART for at least five years and virologically suppressed (undetectable viral load <50 copies/ml). By protocol, these patients perform two or three HIV PCR tests per year. Adherence to therapy is hard to measure, good adherence is achieved when viral load remains suppressed and patients show commitment. HCV and/or HBV co‐infected patients were excluded. Patients were matched by age and sex with HIV‐negative healthy donors (HD) recruited from participating hospitals, with a negative serology HIV test. The study was approved by the ethics committees of the participating hospitals. Written informed consent was obtained from all HIV participants before inclusion in CoRISpe and from all volunteers before inclusion in the study. Data were collected from September 2019 to September 2020.

### 2.2 Laboratory determinations

Cryopreserved peripheral blood mononuclear cells (PBMCs) were provided by the Spanish HIV Hospital Universitario Gregorio Marañón BioBank (HIV HUGM BioBank) [22]. Thirty‐millilitre samples of fresh whole blood from HD volunteers were collected in ethylene diamine tetra‐acetic acid tubes. The percentage of CD4+ and CD8+ T cells was determined in fresh whole blood using Cytomics FC (Beckman‐Coulter, Brea, CA, USA). Plasma and PBMCs were immediately isolated by Ficoll‐Paque density gradient centrifugation and stored at −20°C and −170°C, respectively, until subsequent analysis of IL‐10 and TNF‐α by ELISA assay (R&D Systems, Minneapolis, MN), high‐sensitivity CRP (hsCRP) (ADVIA Chemistry XPT, Siemens Healthineers) and IL‐6 (COBAS e411, Roche Molecular Systems, Basel, Switzerland). Plasma HIV‐1 RNA concentration was measured using quantitative polymerase chain reaction (COBAS Ampliprep/COBAS Taqman HIV‐1 test, Roche Molecular Systems) according to the provided manufacturer's protocol.

### 2.3 Immunophenotyping of T lymphocytes and NK cells

Immunophenotyping of T lymphocytes and natural killer (NK) cells using multiparametric flow cytometry was performed following this hierarchy order according to sample availability. The number of patients included in each subanalysis for the duration of this work may vary due to this limitation. Briefly, PBMCs were thawed, washed and stained with surface marker antibodies, including cell viability, lineage (CD3, CD4, CD8), maturation (CD45RA, CD27), activation (HLA‐DR, CD38, CD69), IL‐7 receptor (CD127), senescence (CD57) and exhaustion markers (PD1, LAG3, TIGIT, TIM3) for T cells. Those cells were stained with marker antibodies including cell viability, lineage (CD56, CD16), activation (HLA‐DR, CD69), natural cytotoxicity family activating receptor (NKp30), C‐type lectin‐like activating (NKG2D) and inhibiting receptor (NKG2A), maturation (CD57) and the constitutively expressed receptor, TIM3, involved in NK‐cell cytokine secretion for NK‐cell phenotype. Isotype controls were included for CD57, TIM‐3, LAG‐3, TIGIT and PD‐1 in T‐cell analysis and for TIM‐3, NKG2D, NKG2A, NKp30 and CD69 in NK‐cell analysis.

The T‐cell maturation subsets were defined based on the expression of CD45RA and CD27 as naïve (CD45RA+CD27+), central memory (CM; CD45RA−CD27+), effector memory (EM; CD45RA−CD27−) and terminally differentiated (TemRA; CD45RA+CD27−). NK cells were classified in three subsets according to the expression of CD56 (CD56^high^, CD56^dim^, CD16^neg^) and CD16 that define the NK cytotoxic cell subset (CD16^high^). Schematic gating strategies for T and NK cells can be found in Figure S1 and Figure S2, respectively.

Acquisition was carried out in a Gallios flow cytometer (Beckman Coulter). Before acquisition, cells were fixed with 4% paraformaldehyde. At least 1 million events were acquired for each condition. Kaluza 2.0 software (Beckman Coulter) was used for data analysis.

### 2.4 Statistical analysis

Continuous variables were expressed as medians and interquartile ranges (IQR). Categorical variables were expressed as number and percentages. Correlations within immunological markers and with the CD4/CD8 ratio and clinical variables were assessed using Spearman's rank test. Differences between categorical and continuous values were determined using chi‐square and Mann‐Whitney *U*‐tests, respectively. *p*‐Values <0.05 were considered statistically significant. The Statistical Package for the Social Sciences software (SPSS 20.0, Chicago, IL, USA) was used for the statistical analysis. Graphs were generated using GraphPad Prism 5.0 (GraphPad Software, Inc., San Diego, CA, USA).

## 3 RESULTS

### 3.1 Characteristics of studied subjects

General and clinical characteristics of the studied group (HIV, *n* = 32) at sampling and the control group of HIV‐negative donors (HD, *n* = 28) age and sex matched, are shown in Table 1. CD4+ T cells frequency was similar in both groups, but CD8+ T cells and CD4/CD8 ratio were different among groups. Characteristics regarding HIV infection including CD4 Nadir, diagnosis and ART exposure are also shown. Immunological and clinical categories of YWVH have been presented as defined by CDC classification [23]. Of the 12 patients in category C (AIDS), seven patients had HIV encephalopathy and five patients had *Pneumocystis jiroveci* infection. Five patients had more than one AIDS‐defining pathology such as recurrent bacterial infections, pneumonia or cachexia.

**Table 1:** Characteristics of the study groups

	YWVH (*n* = 32)	HD (*n* = 28)	*p*
**Characteristics**			
Age, years	24.4 [22.5 to 28.2]	26 [23.5 to 27]	0.360
Sex, male (%)	12 (37.0)	9 (36)	0.907
% CD4+ T cells	35.5 [32.0 to 41.3]	37.8 [35.1 to 41.6]	0.190
% CD8+ T cells^a^	36 [32.6 to 39.0]	20.3 [17.9 to 22.9]	**<0.001**
%CD4/%CD8 ratio^a^	1 [0.7 to 1.4]	1.79 [1.64 to 2.2]	**<0.001**
nCD4+ cells/mm^3^	794 [599 to 981]	*	*
nCD8+ cells/mm^3^ ^a^	774 [622 to 938]	*	*
nCD4+/nCD8+ ratio^a^	1 [0.8 to 1.2]	*	*
Nadir CD4+ cells/mm^3^	198 [76 to 330]		
Immunological category (%)			
1 (>500 CD4+ cells/mm^3^)	0 (0.0)		
2 (201 to 499 CD4+ cells/mm^3^)	16 (50.0)		
3 (<200 CD4+ cells/mm^3^)	16 (50.0)		
Clinical category (%)			
A	5 (15.6)		
B	15 (46.9)		
C	12 (37.5)		
Age at HIV diagnosis, months	8.5 [3 to 30]		
Age at ART initiation, months	49 [14 to 70]		
Time since ART initiation, years	20 [18 to 23]		
On ART regimen (%)			
NRTI + NNRTI	10 (31.2)		
NRTI + PI	6 (18.8)		
NRTI + INSTI	6 (18.8)		
Other ART regimen	10 (31.2)		
Time under virological control, years	8 [7 to 10]		

*Note*: Values are taken at sampling timepoint. All results correspond to median [IQR] except otherwise specified. Continuous variables are expressed as the medians and IQR. Categorical variables are expressed as numbers and percentages. Percentages have been rounded per convention. Mann‐Whitney *U*‐test and chi‐square test were used. Abbreviations: ART, antiretroviral treatment; FI, fusion inhibitor; HD, HIV‐negative healthy donors group; II, integrase inhibitor; INSTI, integrase strand transfer inhibitors; IQR, interquartile range; NNRTI, non‐nucleoside analogue reverse‐transcriptase inhibitors; NRTI, nucleoside analogue reverse‐transcriptase inhibitors; PI, protease inhibitors; YWVH, youth with vertically acquired HIV group.

^a^ Data available for 29 YWVH.

* Absolute values for CD4+, CD8+ and CD4+/CD8+ were not performed but HIV group information was available at CoRISpe database.

### 3.2 YWVH showed increased soluble IL‐6 levels, altered maturation profile and increased activation and exhaustion markers in T lymphocytes

**Figure 1:**
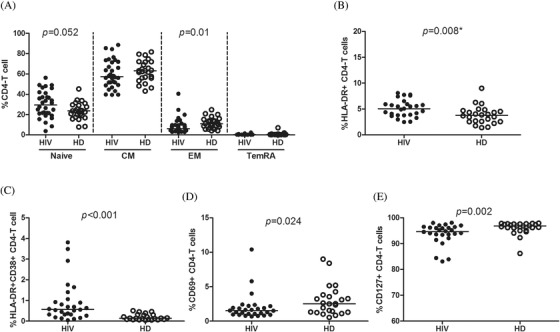
Maturation profile and activation markers on CD4 T cells. Differences in memory subsets distribution **(a)**, activation markers **(b,c,d)** and IL‐7 receptor, CD127 **(e)**. Mann‐Whitney *U*‐test was used to compare groups. HD, group of healthy donors; HIV, group of youths with vertically acquired HIV.

**Figure 2:**
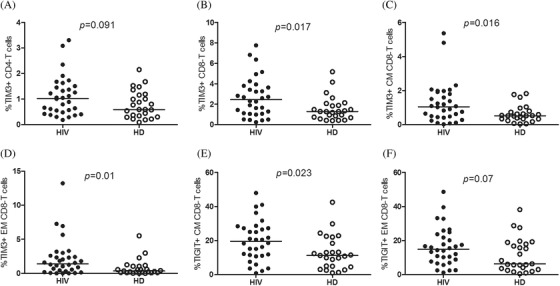
Exhaustion markers on total CD4 T cells and CD8 T‐memory subsets. TIM‐3 expression in total CD4 **(a)** and CD8 T cells **(b)** and TIGIT and TIM‐3 expression in central memory (CM) **(c,d)** and effector memory (EM) **(e,f)** CD8 T cells. Mann‐Whitney *U*‐test was used to compare groups. HD, group of healthy donors; HIV, group of youths with vertically acquired HIV.

Soluble IL‐6 levels were increased in YWVH compared to HD (Table 2). The immunophenotyping analysis of T lymphocytes showed differences in the CD4 T‐cell maturation subsets with increased levels of naïve CD4 T cells and decreased levels of EM CD4 T cells in YWVH compared with HD (Figure 1a). Regarding activation markers, we observed higher expression of HLA‐DR and coexpression of HLA‐DR/CD38 and lower levels of CD69 and IL‐7 receptor expression (CD127) in CD4+ T cells from YWVH when compared with HD (Figure 1b,c,d,e). Similar results were found for those markers in CD8+ T cells, while there were no differences in the maturation profiles (Figure S3). The study of senescence and exhaustion markers in T lymphocytes showed increased levels of TIM‐3 in total CD4+ and CD8+ T cells (Figure 2a,b) and TIGIT and TIM‐3 expression in CM and EM CD8+ T‐cell subsets in YWVH compared with HD (Figure 2c,d,e,f).

**Table 2:** Soluble inflammatory markers

	YWVH (*n* = 32)	HD (*n* = 28)	*p*
hsCRP (mg/dl)	0.11 [0.04 to 0.24]	0.07 [0.02 to 0.14]	0.110
IL‐6 (pg/ml)	4.2 [3.34 to 5.14]	3.29 [2.6 to 3.83]	**0.013**
TNF‐α (pg/ml)	1.49 [1.19 to 1.94]	1 [0.75 to 1.8]	0.131
IL‐10 (pg/ml)^a^	0.31 [0.1 to 0.77]	0.27 [0.17 to 0.39]	0.460

*Note*: Values are taken at sampling timepoint and expressed as the medians and interquartile (IQR) ranges. Abbreviations: HD, HIV‐negative healthy donors group; hsCRP, high‐sensitivity C‐reactive protein; YWVH, youth with vertically acquired HIV group.

^a^ Data available for 21 and 16 YWVH and HD, respectively.

### 3.3 Different NK‐cell subset distribution and high levels of activation and exhaustion markers in YWVH

**Figure 3:**
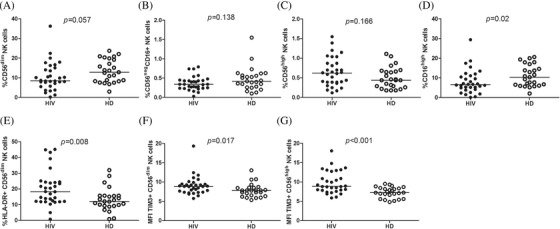
Frequency of NK‐cell subsets, activation and exhaustion markers. Frequency of CD56^dim^, CD56^neg^CD16+, CD56^high^ and CD16^high^ NK‐cell subset **(a,b,c,d)**. HLA‐DR in CD56^dim^ NK cells **(d)** and median‐intensity fluorescence (MFI) of TIM‐3 expression in CD56^dim^ and CD56^high^ NK subset **(e,f,g)**. Mann‐Whitney *U*‐test was used to compare groups. HD, group of healthy donors; HIV, group of youths with vertically acquired HIV.

There was a trend towards a lower frequency of CD56^dim^ and CD56^neg^CD16+ and an increased percentage of CD56^high^ NK subset in YWVH (Figure 3a,b,c). Within the CD56^dim^ subset, the NK cytotoxic cell population CD16^high^ was decreased in YWVH (*p* = 0.02) (Figure 3d). Interestingly, we observed increased levels of HLA‐DR in CD56^dim^ and MFI of TIM‐3 expression in CD56^dim^ and CD56^high^ NK subsets (Figure 3e,f,g).

### 3.4 Activation and exhaustion markers from T cells and NK‐cell subsets correlated with CD4/CD8 ratio, CD4± nadir and age at ART initiation in YWVH

**Figure 4:**
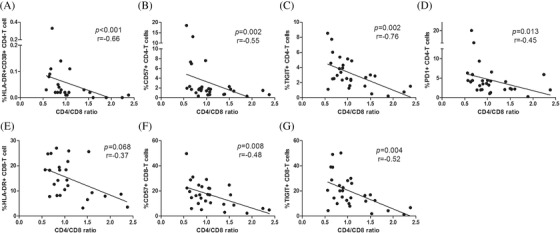
CD4/CD8 ratio correlations with CD4 and CD8 T‐cell activation and exhaustion levels in YWVH. Correlations of ratio CD4/CD8 and HLA‐DR CD38, CD57, TIGIT and PD‐1 expression in total CD4 **(a,b,c,d)** and HLA‐DR, CD57 and TIGIT expression in CD8 T cells **(e,f,g)**. The Spearman *ρ* correlation coefficient test was used.

We then studied the relation between activation and exhaustion markers in the adaptive and innate immune components with the CD4/CD8 ratio in the group of YWVH. To analyse the impact of different ART regimens on immunity was not possible due to the highly heterogeneity of therapies received by our patients. The expression of activation markers (HLA‐DR and CD38) and exhaustion (CD57, TIGIT and PD‐1) in total CD4+ T cells strongly and inversely correlated with the CD4/CD8 ratio (Figure 4a,b,c,d). Similar associations were found for total CD8+ T cells for HLA‐DR, CD57 and TIGIT expressions (Figure 4e,f,g). In addition, the same analysis split by T‐cell memory subsets also showed significant correlations for CM and EM CD4+ T‐cell subsets and TemRA CD8+ T‐cell memory subset (data not shown).

**Figure 5:**
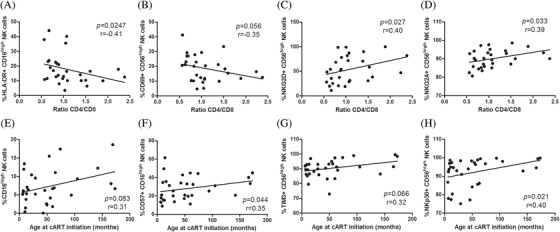
Correlations between NK activation and exhaustion levels and CD4/CD8 ratio and age at ART initiation in YWVH. Correlations of ratio CD4/CD8 with HLA‐DR expression in CD16^high^ NK subset **(a)**, CD69, NKG2D and NKG2A in CD56^high^ NK subset **(b,c,d)**. Patient age at ART initiation (months) correlations with CD16^high^ NK subset levels and CD57 expression **(e,f)** and TIM‐3 and NKp30 expression in CD56^high^ NK subset **(g,h)**. The Spearman *ρ* correlation coefficient test was used.

Regarding NK cells of YWVH, CD69 expression in all NK‐cell subsets inversely correlated with the ratio CD4/CD8. Direct correlations were found for NKG2D expression in all subsets and ratio CD4/CD8 also correlated inversely with HLA‐DR and directly with NKG2D in CD56^high^, CD56^dim^ and CD16^high^ NK‐cell subsets (representative graphs are shown in Figure 5a,b,c,d). Besides, CD57 expression in CD56^neg^CD16+ subset also correlated inversely with ratio CD4/CD8 (*r* = −0.43; *p* = 0.018). We also found direct associations among the age at ART initiation and the frequency of CD16^high^ NK‐cell subset (Figure 5e), the expression of CD57, TIM3 and NKp30 markers in CD56^high^, CD56^dim^ and CD16^high^ NK‐cell subsets (representative graphs in Figure 5f,g,h).

Interestingly, the time under ART correlated with HLA‐DR, NKG2D and NKp30 expression (*r* = 0.4, *p* = 0.025; *r* = −0.4, *p* = 0.026; *r* = −0.42, *p* = 0.016, respectively) not only in CD16^high^ subset (Figure S4a,b,c) but also in CD56^high^ and CD56^dim^ subsets (data not shown). In the case of CD56^neg^CD16+ subset, CD69 and CD57 expression was also correlated with the time under ART (*r* = 0.38, *p* = 0.036 and *r* = 0.51, *p* = 0.003, respectively). Moreover, CD4 nadir was also correlated with CD69, NKG2D and TIM‐3 expression in CD56^high^ NK cells (*r* = −0.49, *p* = 0.004; *r* = 0.48, *p* = 0.005; *r* = 0.42, *p* = 0.015, respectively) and with CD69 in CD56^neg^CD16+ NK cells (*r* = −0.35, *p* = 0.049) (Figure S4d,e,f,g).

## 4 DISCUSSION

Our results show that the adaptive and innate immune system of YWVH is characterized by an increased activation and exhaustion profile, even after long‐term suppression under ART. The CD4/CD8 ratio is strongly correlated with markers of activation and exhaustion of the immune system in this population, as well as age at ART initiation, or low CD4 nadir. This study demonstrated that immune activation and exhaustion persist even after several years of ART.

The evidence suggests that the immune dysfunction plays a crucial role in HIV pathogenesis, leading to non‐AIDS morbidity and mortality in PLWH [24, 25, 26, 27, 28, 29]. First children infected at the beginning of the HIV pandemic are now reaching adulthood, but studies regarding the evolution of the immune system of YWVH are rather scarce. Of our 38 participants, only one of them started treatment with highly active antiretroviral therapy (HAART) as first antiretroviral therapy, the rest started treatment with suboptimal treatments, which could explain the long time to achieve viral suppression. There could be other causes such as bad adherence to therapy, which is very difficult to measure in our YWVH population, a complicated cohort with losses to follow‐up and poor retention in care.

In our study, YWVH had increased IL‐6 levels, well known to be associated with high‐morbidity risk [30] and, focusing on immune phenotype, maturation profile was altered among CD4 T cells, resulting in an increase in naïve and low central and effector memory subsets in YWVH. We also observed increased activation (HLA‐DR and CD38) and exhaustion markers on T cells compared to uninfected donors in concordance with previous studies [12, 13]. Our results suggest that immune activation persists in YWVH, even among those on ART and virologically suppressed. These results contrast with previous results suggesting that treated and suppressed patients had a T‐cell activation and immune exhaustion profile similar to uninfected adolescents [31]. Distinct mention deserves results obtained for CD69 on T cells, considered the earliest activation marker during infections and increased levels have been associated with increased viral load [32, 33]. However, scarce evidence described up to now was on HIV patients infected at adulthood and naïve for ART. The early HIV diagnosis and long‐suppressed viral load of our studied group of HIV young adults could explain the lack of accordance with the previous observations, and the persistent and long use of ART could explain the CD69 decreased levels compared with HD.

Features of immunosenescence in HIV‐negative individuals include low CD4/CD8+ T‐cell ratio [34, 35]. In most PLWH, ART normalizes CD4/CD8 ratio by increasing the percentage of CD4+ T cells to normal levels [28, 36, 37, 38, 39]. However, a persistent low ratio has been described in certain patients despite ART and is thought to be associated with immunological abnormalities. Previous studies have suggested that the CD4/CD8 ratio is a valuable marker of ageing, immunosenescence and even mortality [40, 41]. We explored the association between activation and exhaustion expression markers on both CD4+ and CD8+ T cells, and found that they strongly and inversely correlated with CD4/CD8 ratio in our YWVH population. Even our results suggest that blocking these targets (TIM3, PD1 and TIGIT) could slow down T‐cell exhaustion and restore CD4/CD8 ratio [42], and the use of these checkpoint inhibitors might also result in adverse events [43, 44].

Innate immune system is critically important at the onset of any infection but has barely been studied in YWVH. Recent innovative therapeutics and vaccine strategies are nowadays focused on this arm of immunity [45, 46]. NK cells play a primary role in the innate immune response by targeting virally infected and transformed cells through direct killing and providing help to adaptive responses through cytokine secretion. A chronic NK‐cell activation phenotype has been described in YWVH [47, 48]. In our study, differences among NK‐cell subset distributions were found: the cytotoxic subset CD16^high^ NK cells decreased in YWVH compared to uninfected controls, as previously described [49]. Some studies of children with vertically acquired HIV‐1 observed differences in the NK‐cell subsets and functions [50], and our results are in line with previous observations carried out in PLWH that suggest a defective NK‐cell‐mediated cytotoxic activity [51]. HLA‐DR and TIM3 expressions are not limited to T cells. According to our results, the level of both markers on NK cells are dysregulated in YWVH and this fact could lead to increased cytokine production and degranulation, which could impair an antiviral response [42]. NK‐cell subset distribution and the expression of activating and inhibiting receptors (NKp30, NKG2A and NKG2D) show strong association with the CD4/CD8 ratio and other infection‐related parameters (i.e., age at treatment initiation, time on ART and CD4+ T‐cell nadir). These observations agree with previous observations on PLWH whose NK‐cell markers correlated with indicators of disease severity such as decreased CD4‐T‐cell counts and plasma viral load [52, 53, 54, 55]. These facts reinforce the idea of exhaustion markers blockage as a target for immunomodulatory therapy in HIV infection. However, the unexpected and discordance behaviour observed in some correlations of markers, such as NKG2D, TIM3 and NKp30, generally upregulated and associated with HIV disease progression may reflect a dysregulated immune system characteristic of this YWVH population. Several factors may underlie these findings. First, the early infection of our patients when their immune system was still immature. Second, long exposure to ART, with very old forms of treatments, which were not always effective in achieving viral suppression. Last, chronic exposure of an immature immune system to persistent chronic inflammation could have a deleterious effect on the innate immune system. Exposure to HIV without treatment probably leads to a greater level of inflammation and immune activation, and consequent loss of function of effector cells. This hypothesis may explain the strong associations found between NK‐cell markers and the age at ART initiation in our study, in which most patients had started treatment rather late compared to today's standards. Therefore, this may not be the same for children, as they now receive ART from birth.

The small sample size is the main limitation of this study. However, the main strength is the selection of youths acquiring HIV early in their life who are now adults. As a demographic, it is yet to be thoroughly studied. To address the usefulness of the CD4/CD8 ratio to identify subjects at risk of adverse events, a longitudinal design would be needed, as it appears that our participants might be too young and therefore we do not have any evidence of a higher prevalence of cardiac, metabolic, endocrine, gastrointestinal or psychiatric diseases at this moment. Nevertheless, the prospective follow‐up of the CoRISpe cohort would help us to find possible future associations between activation markers and comorbidities.

## 5 CONCLUSIONS

Results of our study highlight the importance of early treatment in YWVH, as age at treatment initiation; time under ART and a low CD4 nadir are related to activation/exhaustion of the immune system. However, our results suggest that innate and adaptive immune dysfunction persist in YWVH despite ART. Our results support the use of the CD4/CD8 ratio as a marker of a dysregulated immune system in YWVH in clinical practice.

## COMPETING INTERESTS

The authors declare that they have no competing interests.

## AUTHORS’ CONTRIBUTIONS

I.C., L.T., E.V. and A.Z. performed the research. M.A.M.F and M.L.N. designed the research study. S.J. contributed essential tools. I.C, L.T. and E.V. analysed the data. I.C. and L.T. wrote the paper. E.V., S.J., T.S., M.A., C.E., S.G., J.T.R., C.D., J.I.B., J.A.I., M.A.M.F. and M.L.N. critically discussed the paper. All authors have read and approved the final manuscript.

## FUNDING

This work was supported by the Acción Estratégica en Salud, Plan Nacional de Investigación Científica, Desarrollo e Innovación Tecnológica [Grant Number PI19/01530]. L.T. was funded by Acción Estratégica en Salud, Plan Nacional de Investigación Científica, Desarrollo e Innovación Tecnológica (2013‐2016) [Grant Number RD16/0025/0019] and cofinanced by Instituto de Salud Carlos III (Subdirección General de Evaluación) and Fondo Europeo de Desarrollo Regional (FEDER). E.V. was supported by RETIC [Grant Number PT17/0015/0042] and Programa Operativo de Empleo Juvenil financed by Fondo Social Europeo [PEJ‐2017‐AIBMD‐7446].

## Supporting information

**Figure S1**. Schematic diagram of lymphocyte gating strategy of a healthy donor

**Figure S2**. NK cells and subset gate strategy (A) and histogram representation of markers expression on CD56+CD16^high^ subset (blue) compared to each isotype control (red) (B)

**Figure S3**. Activation and maturation profile on CD8‐Tcells

**Figure S4**. NK activation and exhaustion markers correlations with the time under cART and nadir CD4
