# Supplementary material for: Innate and adaptive abnormalities in youth with vertically acquired HIV through a multicentre cohort in Spain
Source: J Int AIDS Soc. 2021 Oct 20;24(10):e25804. doi: 10.1002/jia2.25804 (PMC8528666; doi:10.1002/jia2.25804)
Supplement: Supplementary file 1 [file JIA2-24-e25804-s005.docx]

**Supplementary material:**

For T lymphocyte immunophenotyping, two flow cytometer panels were designed. For the first panel PBMCs were thawed, washed with PBS containing 3% of Bovine Serum Albumin (BSA) (Millipore) and stained for 30 minutes with LIVE/DEAD fixable Aqua Blue Dead Cell Stain (Life Technologies, CA, USA) for viability, and the surface antibodies CD56-CD19-CD14-BV510 (CD56 clone NCAM16.2; CD19 clone SJ25C1 and CD14 clone MOP9), CD3-PerCP-Cy5.5 (clone SK7) (BD Biosciences, San Diego, CA) and CD8-PB (clone RPA-T8; Biolegend, San Diego, CA) for lineage, CD45RA-ECD (clone 2H4), CD127-PeCy7 (clone R34.34), HLA-DR-APC (clone GRB-1) (Beckman Coulter), CD38-FITC (clone HB7), CD69-APC-R700 (clone FN50) (BD Biosciences) for maturation, survival and activation.

The second panel for T lymphocyte included: LIVE/DEAD fixable Aqua Blue Dead Cell Stain for viability, CD56-CD19-CD14-BV510 (CD56 clone NCAM16.2; CD19 clone SJ25C1 and CD14 clone MOP9), CD3-APC-Cy7 (clone SK7) and CD4-APC-R700 (clone RPA-T4, BD Biosciences) for lineage, CD45RA-ECD (clone 2H4; Beckman Coulter) and CD27-PerCP-Cy5.5 (clone M-T271, BD, Biosciences) for maturation, CD57-FITC (clone NC1; Beckman Coulter) for senescence and TIM-3-PE (clone 7D3), PD-1 (clone EH12.1) (BD Biosciences), LAG-3-Pe-Cy7 (clone 7H2C65) and TIGIT-AF647 (clone A15153G) (Biolegend), for exhaustion. Lymphocytes were defined as viable cells having low forward/side scatter and expressing CD3, and/or no CD4, but not CD19, CD14 and CD56. Isotypes controls were included for CD57, TIM-3, LAG-3, TIGIT and PD-1 (**Supplementary figure 1 A-B**).

The NK immunophenotyping included: LIVE/DEAD fixable Aqua Blue Dead Cell Stain for viability, CD3-CD19-CD14-BV510 (CD3 clone SK7; CD19 clone SJ25C1 and CD14 clone MOP9), CD56-APC-Cy7 (clone NCAM16.2), CD16-PerCP-Cy5.5 (clone 3G8) for NK subsets identification (BD Biosciences) and CD57-FITC (clone NC1), HLA-DR-APC (clone GRB-1) (Beckman Coulter), TIM-3-PE (clone 7D3), NKG2D-PECF594 (clone 1D11), CD69-APC-R700 (clone FN50), NKG2A-BV421 (clone 131411) (BD Biosciences) and NKp30-PE-Cy7 (clone P30-15; Biolegend), for activation, cytotoxic activity, maturation and activating and inhibiting receptor expression. To identify NK cell, viable cells, negative for CD3, CD14 and CD19 were classified according to the expression of CD56 and CD16: CD56^high^, CD56^dim^ that includes CD16^high^ subset, and CD56^neg^CD16+. Isotype controls were included for TIM-3, NKG2D, NKG2A, NKp30 and CD69 (**Supplementary figure 2 A-B**).

Acquisition was carried out in a Gallios flow cytometer (Beckman Coulter). Before acquisition, cells were fixed with 4% PFA. At least 1 million events were acquired for each condition.

**Supplementary figure 1.** Schematic diagram of lymphocyte gating strategy of a healthy donor. Lymphocyte gate strategy, co-expression of CD38 and HLA-DR and memory subset distribution based on CD27 and CD45RA expression (A). Histogram representation of activation, senescence and exhaustion markers expression on CD8 T-cells (blue) compared to each isotype control (red).

**Supplementary figure 2.** NK cells and subset gate strategy (A) and histogram representation of markers expression on CD56+CD16^high^ subset (blue) compared to each isotype control (red) (B).

**Supplementary figure 3.** Activation and maturation profile on CD8-Tcells. Differences in activation markers (A-C) and IL-7 receptor marker, CD127 (D) on total CD8 T-cells. Representation of CD8 T-cell memory subsets distribution (E). Mann-Whitney U-test was used to compare groups.

- HIV= group of youths with vertically acquired HIV
- HD= group of healthy donors

**Supplementary figure 4.** NK activation and exhaustion markers correlations with the time under cART and nadir CD4. Time under cART (years) correlations with the of HLA-DR, NKG2D and NKp30 expression in CD16^high^ NK subset (A-C) and nadir CD4 counts (cells/mm^3^) correlations with CD69, NKG2D and TIM-3 expression in CD56high NK subset (D-F) and CD69 expression in CD56negCD16+ NK subset (G) in youths with vertically acquired HIV. The Spearman ρ correlation coefficient test was used.
