# Supplementary figures and images for: Innate and adaptive abnormalities in youth with vertically acquired HIV through a multicentre cohort in Spain
Source: J Int AIDS Soc. 2021 Oct 20;24(10):e25804. doi: 10.1002/jia2.25804 (PMC8528666; doi:10.1002/jia2.25804)

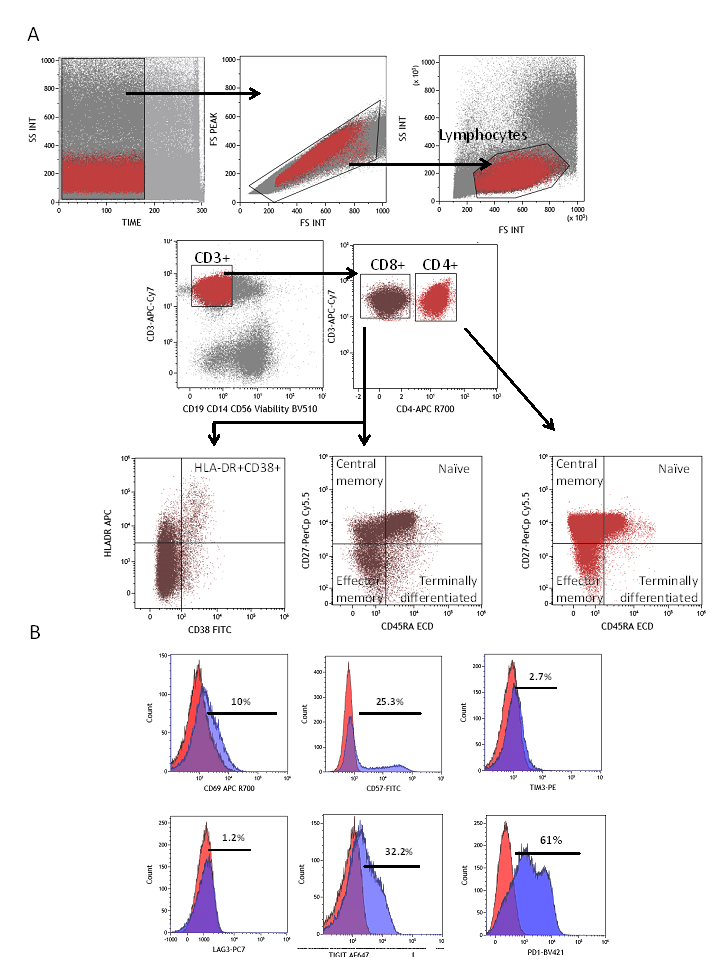

Supplement: Supplementary file 2 — Figure S1. Schematic diagram of lymphocyte gating strategy of a healthy donor [file JIA2-24-e25804-s004.tif]

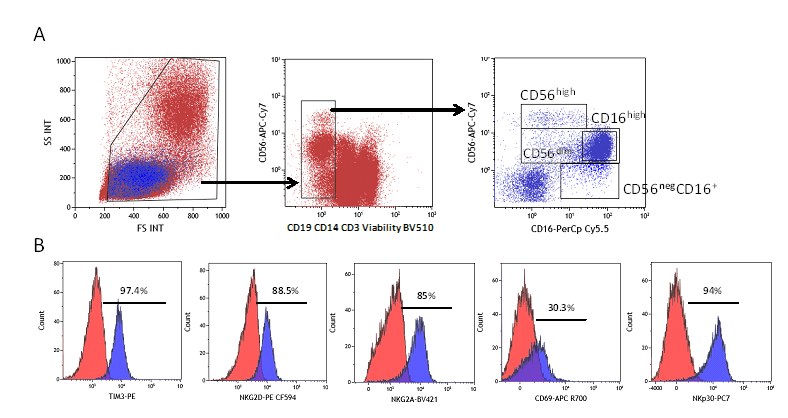

Supplement: Supplementary file 3 — Figure S2. NK cells and subset gate strategy (A) and histogram representation of markers expression on CD56+CD16high subset (blue) compared to each isotype control (red) (B) [file JIA2-24-e25804-s003.tif]

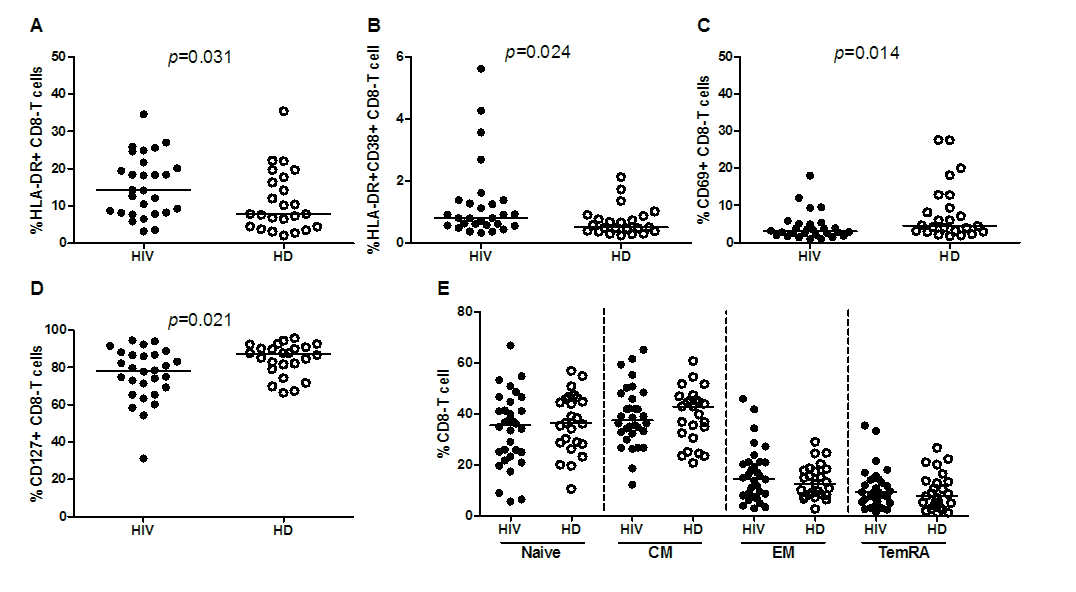

Supplement: Supplementary file 4 — Figure S3. Activation and maturation profile on CD8‐Tcells [file JIA2-24-e25804-s002.tif]

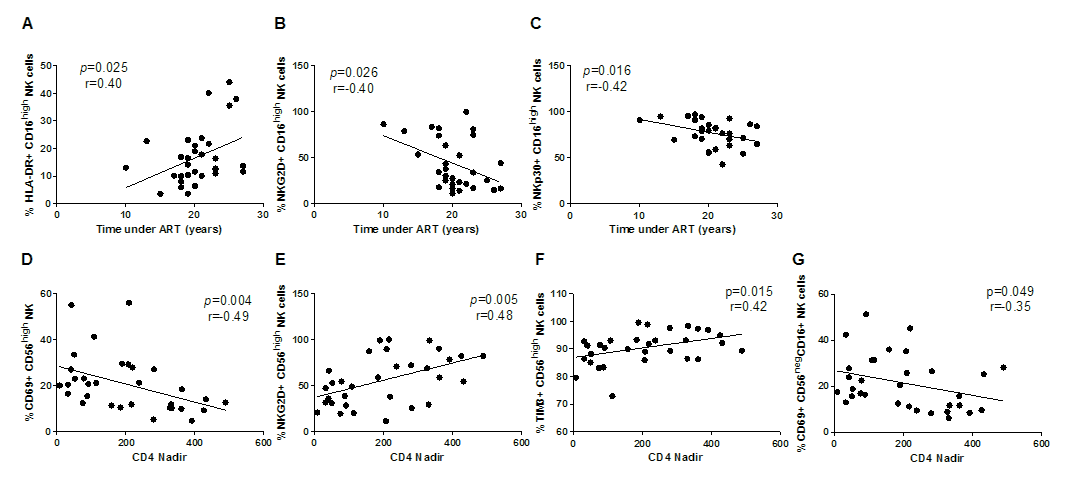

Supplement: Supplementary file 5 — Figure S4. NK activation and exhaustion markers correlations with the time under cART and nadir CD4 [file JIA2-24-e25804-s001.tif]
